# Phrenoplasty Techniques for the Reconstruction of Basal Chest Wall Defects

**DOI:** 10.3390/jcm13195928

**Published:** 2024-10-04

**Authors:** Francesco Puma, Silvia Ceccarelli, Alberto Melis, Domenico Pourmolkara, Eleonora Coviello, Riccardo Amatucci, Niccolò Daddi, Jacopo Vannucci

**Affiliations:** 1 Department of Thoracic Surgery, University of Perugia Medical School, Ospedale Santa Maria della Misericordia, 06123 Perugia, Italy; fr_puma@yahoo.it (F.P.); silvia1.ceccarelli@ospedale.perugia.it (S.C.); melis.alberto@outlook.it (A.M.); domenico.pourmolkara@gmail.com (D.P.); eleonoracoviello@hotmail.it (E.C.); riccardo.amatucci@ospedale.perugia.it (R.A.); 2 Department of Thoracic Surgery, University of Bologna “Alma Mater Studiorum”, Ospedale S. Orsola, 40126 Bologna, Italy; niccolo.daddi@unibo.it; 3 Department of Thoracic Surgery and Lung Transplantation, University of Rome Sapienza, Policlinico Umberto I, 00185 Rome, Italy

**Keywords:** chest wall tumors, chest wall surgery, surgical technique

## Abstract

Background: Primary and secondary tumors of the abdominal lower third of the bony thorax are relatively rare. Therefore, indications and techniques for chest wall reconstructions in this area are not well defined. Methods: The techniques for reconstructing basal chest wall defects using the diaphragm are described. Indications for phrenoplasty are limited to reconstruction after full-thickness resection of at least two of the last four ribs in the midaxillary line. The diaphragm can be used for reconstructive purposes both if it is intact and if it is partially involved in the resection of the chest wall. Results: At our institution, the abovementioned reconstructive technique was successfully performed in five patients with an uneventful post-operative course. Conclusions: The main advantages of these methods are the use of promptly available, high-quality autologous tissue and the exclusion of the pleural space from the defect area, thus transforming a thoracic defect into an abdominal one. The disadvantage is a variable reduction in the volume of the hemithorax. These techniques could be compared with other reconstruction techniques using pre-/post-operative respiratory functional tests.

## 1. Introduction

The lower third of the rib cage is an anatomical transition area between the thorax and the abdomen. This area is rarely resected for malignancy. Removal of two or more consecutive lower midaxillary ribs results in a basal defect corresponding to the costophrenic recess. Depending on the location and extent of the tumor, resection may involve the diaphragm, thus creating direct thoracoabdominal communication. The reconstructive planning, which may involve possible specific technical problems, is aimed at ensuring preservation of the ventilatory function, repair of the diaphragm and protection of the thoracic and abdominal viscera. The diaphragm can be used for reconstructive purposes in selected patients, thus representing a simple solution to challenging cases [1]. Systematic studies on the possibility of using the diaphragm as a source of tissue to reconstruct chest wall defects have not been performed, considering the epidemiology and therapeutic indications. However, there is more than one report presenting the diaphragm as a suitable organ to restore basal chest wall defects. In this study, the techniques of chest wall reconstruction with the diaphragm after tumor resection are described in view of offering potential alternative options to have in the armamentarium whenever the diaphragm is more accessible than other muscles. To our knowledge, phrenoplasty with the present technique for this particular area of the chest has never been described before.

## 2. Techniques

**Figure 1:**
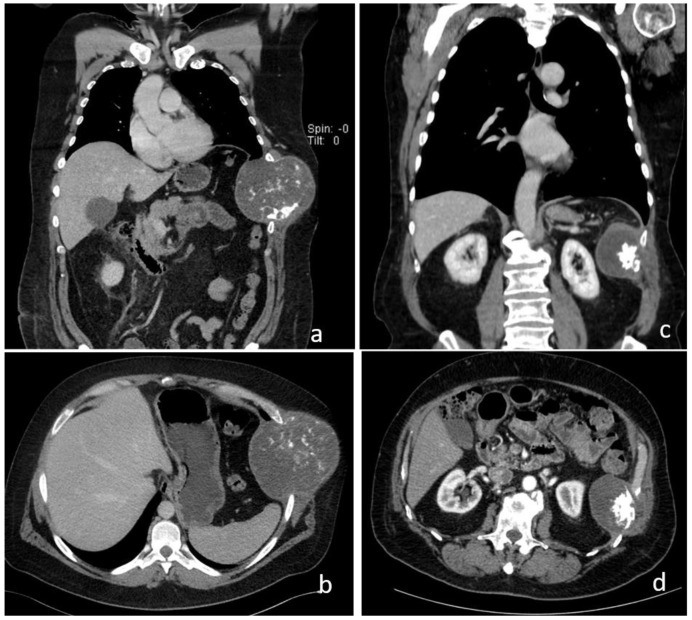
Computed tomography findings of two patients with primary chondrosarcoma of the chest wall in coronal (**a**,**c**) and axial reconstruction (**b**,**d**). Tumor involving the 7th and 8th ribs (**a**,**b**). Tumor involving the 9th and 10th ribs (**c**,**d**).

Two different techniques of phrenoplasty can be utilized based on whether the diaphragm has been partially resected or not. Indications for these techniques are uncommon and reserved for the repair of chest wall defects resulting from resection of at least two consecutive ribs among the 7th, 8th, 9th and 10th ribs at the midaxillary line (Figure 1).

### 2.1. Phrenoplasty with Intact Diaphragm

**Figure 2:**
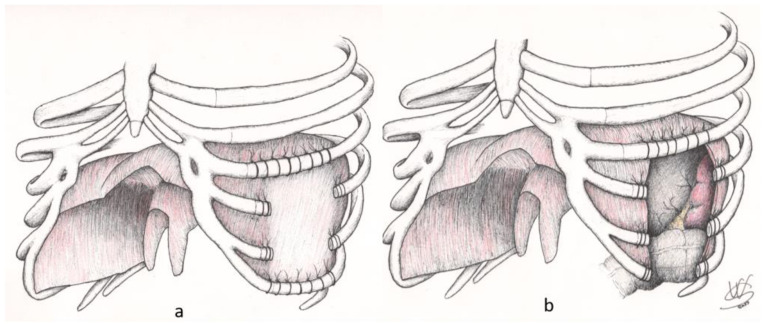
Phrenoplasty techniques for reconstruction of basal chest wall defects: The diaphragm is not involved in chest wall resection (**a**). The diaphragm is involved in chest wall resection (**b**).

**Figure 3:**
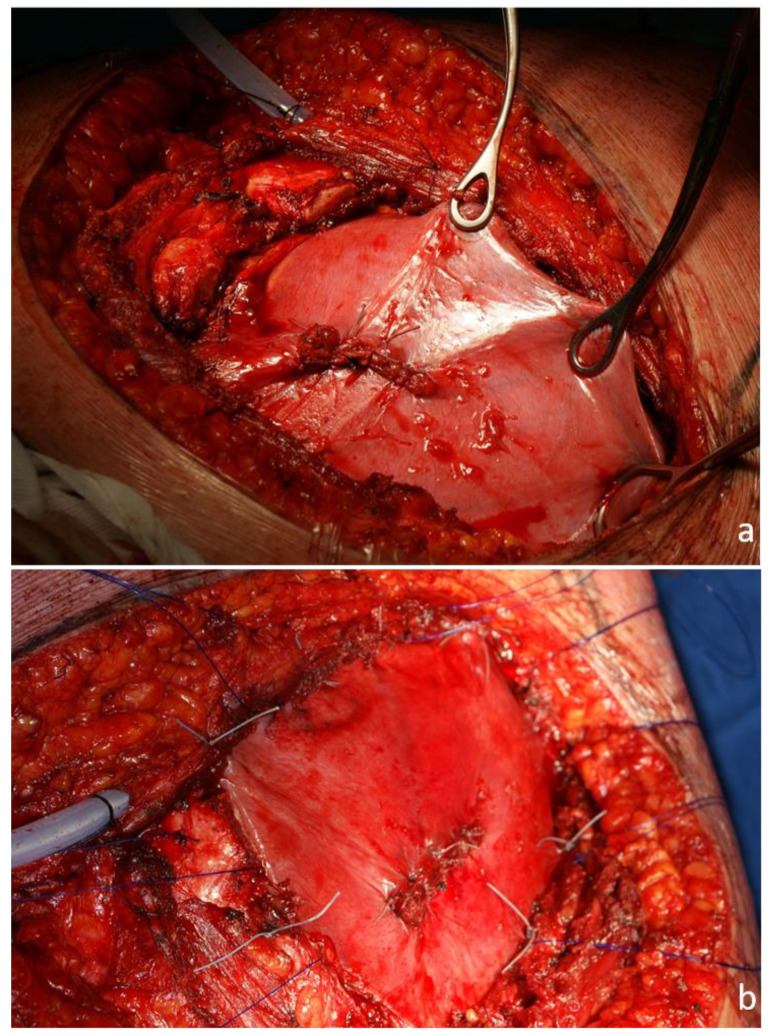
Intraoperative view. Resection of the 7th and 8th ribs without involving the diaphragm. Full-thickness defect in the area of the costophrenic recess (**a**). The repair is performed by suturing the diaphragm circumferentially at the edges of the defect (**b**).

The diaphragm is not always involved in inferiorly located chest wall tumors, especially when they arise from the 7th and 8th ribs. In these cases, the defect faces the descending portion of the diaphragm dome and is located at the bottom of the pleural cavity. A simple and effective repair of these chest wall defects is conducted by fixing the adjacent diaphragm to the edges of the defect (Figure 2a). The diaphragm is grasped with tissue-holding forceps (Figure 3a) and circumferentially sutured with heavy non-absorbable stitches to the intact upper and lower ribs, to the stumps of the resected ribs and to the intercostal soft tissues (Figure 3b). An additional wall reinforcement with a mesh prosthesis is an option to be reserved only for large defects involving three or more ribs. According to our experience, the highest level to possibly anchor the diaphragm without using a mesh is potentially the 6th rib in the lateral segment. This is not a dogma but strictly depends on the case.

### 2.2. Phrenoplasty with Detachment of the Costal Diaphragmatic Insertions

**Figure 4:**
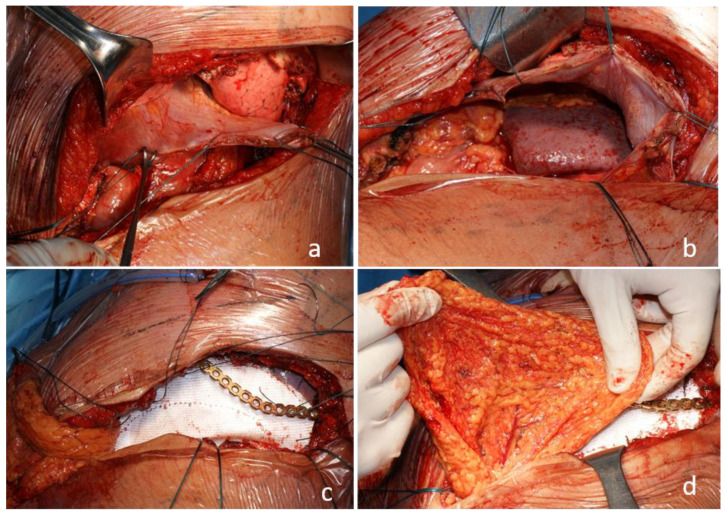
Intraoperative view. Resection of the 9th and 10th ribs involving the diaphragm. The chest wall en bloc with the lateral diaphragm is resected. The diaphragm is prepared for translation towards the 8th rib as a pedicled muscle flap. The stitches serve as temporary grasps to better fashion the necessary flap (**a**). The new diaphragm is used as a muscle flap to divide the chest and the abdomen, excluding the defect from the pleural cavity. The diaphragm is sutured to the 8th rib with single stitches (**b**). The abdominal defect is repaired/reinforced with a PTFE mesh, and a titanium bar is positioned to replace one costal arch (**c**). The omental flap is used to fill the gap with autologous tissue and to reinforce the local vascular supply (**d**).

A chest wall tumor affecting the ribs across the diaphragmatic insertions requires a slightly more demanding reconstruction procedure. With the peripheral resection of the diaphragm, the pleural and abdominal cavities are connected. In addition to the stabilization of the thoracoabdominal wall, reconstruction involves the issue of separating the thorax from the abdomen through the repair of the diaphragm. The latter must be reattached somewhere to the wall and must retain its function as far as possible. Therefore, the focus of the reconstruction plan is on how to repair the diaphragm and at what level to reattach it. Detachment of the diaphragm from its costal insertions creates a muscle flap (Figure 4a) that is easily fixable to the intact rib at the upper edge of the defect (Figure 4b), thus transforming the thoracic defect into an abdominal one (Figure 2b). The only condition for this procedure is that the diaphragm resection should be peripheral and not too extensive. The pleural volume reduction is commensurate with the rib to which the flap is attached, although it occurs primarily in the costophrenic recess. Anchoring the muscle above the level of the 6th rib is not recommended because the higher the diaphragm fixation, the greater the thoracic shape modification and pleural volume reduction. Once the pleural cavity is sealed and topographically excluded from the area of resection, the residual abdominal wall defect is generally repaired to ensure protection for the abdominal organs and minimize the risk of abdominal hernia. The kind of reconstruction and the type of materials suitable for this final step depend on the extent of the defect and the choice of the surgeon. Titanium bars and synthetic meshes are reasonable choices (Figure 4c); but, whichever reconstructive technique is chosen, the omentum can be useful as it is promptly available, reinforces the local vascular supply and does not require an additional incision (Figure 4d).

## 3. Results

**Figure 5:**
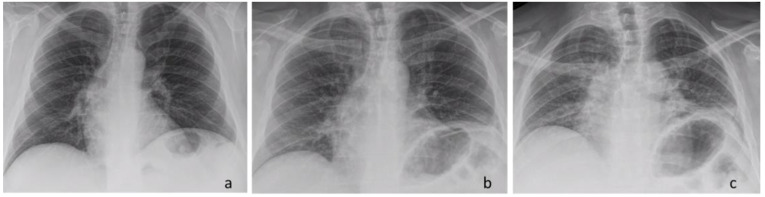
Chest X-ray: Pre-operative finding—inspiration view (**a**). Post-operative finding—inspiration view (**b**). Post-operative finding—expiration view (**c**).

**Figure 6:**
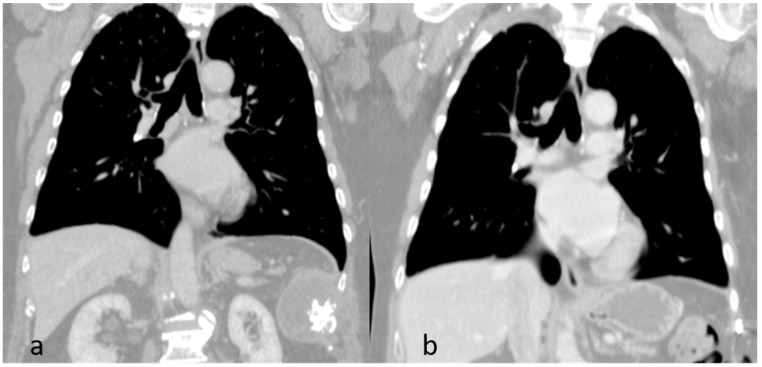
Coronal view computed tomography scans: Pre-operative finding (**a**). Post-operative finding (**b**).

Over a period of twenty years, we performed phrenoplasty techniques on five patients (four for chondrosarcomas and one for a huge single bony relapse from lung adenocarcinoma) [2]. In three patients, the diaphragm was not involved in the resection, and we avoided bony reconstruction (only two ribs were resected). In the remaining two patients, the diaphragm flap was fixed to the uninvolved lower rib, and the rib cage was reconstructed with synthetic meshes, titanium bars and omentum. Diaphragm function is uncertain when the muscle is reattached to a higher rib due to its subsequent flattening. However, if its innervation and blood supply have been preserved, the muscle may retain some contractile capacity, as demonstrated by inspiratory and expiratory chest X-rays (Figure 5 and Figure 6). These procedures are obviously followed by some radiological sequelae, which are the result of the anatomical modifications induced both by the resection and by the reconstruction: blunting of the costophrenic recess, moderate reduction in the volume of the pleural cavity and slight flattening of the diaphragm, whose excursion is reduced but not prevented.

No patient had post-operative complications or early or late ventilatory problems. The patient who underwent the most extensive resection of the diaphragm and chest wall (7th, 8th, 9th and 10th ribs) had an asymptomatic, late weakness of the abdominal wall that did not require surgical revision (we cannot specifically disclose whether it was a true hernia or a disembowelment due to an oblique muscle paralysis for the intercostal nerve section). Two patients resected for chondrosarcoma are still disease-free, while the other two died of other causes 8 and 13 years after surgery, respectively. The patient operated on for lung adenocarcinoma died of metastatic disease after 7 months. No patients had decreased quality of life due to respiratory problems.

## 4. Discussion

The lower ribs provide attachments to the diaphragm and offer bony protection for the splanchnic organs, while they are only marginally located in the pleural space. The lower third of the bony thorax is rarely affected by adjacent malignant tumors, as T3-lung cancer usually infiltrates the upper or mid-thoracic ribs [3], and invasion of the chest wall by breast tumors does not occur in this area. For this reason, resection and reconstruction of this topographic area of the chest wall are uncommon and are indicated mainly for primary tumors. 

Although the lower rib cage is predominantly abdominal [4], the intrapleural forces strongly act at this level due to the contraction of the diaphragm; in fact, during the breathing cycle, these ribs undergo a significant excursion since they move laterally when elevated [1]. Therefore, wide basal chest wall defects elicit a significant post-operative paradoxical chest movement, requiring surgical stabilization.

This chest wall area involves reconstructive techniques after tumor resection that are not well codified in the literature and are mostly related to the variable local situation. The concept of simply attaching the diaphragm at a higher level was described in the 1950s to reduce dead space after lobectomy [5], but such a method did not find widespread use as it was deemed too invasive for the purpose.

The application of this technique for the repair of lower chest wall defects after tumor resection was revived by an Italian surgeon in the 1980s [6], and it was occasionally reported in some case series [2,7]; however, indications, technical details and potential advantages have never been analyzed.

The question of possible diaphragm involvement in the resection of caudally located chest wall tumors opens up different clinical scenarios regarding both the anatomical impairment and the consequent reconstructive issues. Reconstructive techniques are, therefore, diversified on the basis of the possible infiltration of the diaphragm by the tumor.

Lower chest wall resection not involving the diaphragm may be effectively repaired by the diaphragm, which can be circumferentially sutured to the margins of the wall defect, thus moving the defect into an extrathoracic area and eliminating any paradoxical movement of the chest wall. In our opinion, the simple reconstruction of the chest wall with a prosthetic or biological mesh has no advantages over this kind of phrenoplasty; in fact, the obliteration of the costodiaphragmatic recess is inevitable for defects located in this area regardless of the reconstruction technique, and the diaphragm function seems not to be further impaired by the phrenoplasty procedure itself. On the other hand, the latter allows immediate and reliable chest wall stabilization without using prosthetic materials, although a chest wall mesh repair could be added to further reinforce the reconstruction of very large defects.

Tumors requiring a lower chest wall resection en bloc with the peripheral diaphragm involve the added problem of restoring the separation between the chest and the abdomen. Reconstruction should be customized based on the extent of the defect in the chest wall and diaphragm. Kuwahara et al. [8] recommend repairing limited diaphragm resections (up to 3–4 cm) by pulling the muscle to its original position. However, since the ribs where the diaphragm was originally inserted have been resected, the diaphragm flap must be anchored to the chest wall mesh [9]. For larger diaphragm resections, a double-mesh reconstruction has been advocated, with the diaphragm mesh sutured to the thoracoabdominal wall reconstruction mesh. Even this reconstruction does not seem ideal, as it entails suturing together two meshes subjected to tension at different points. In these cases, phrenoplasty seems to be a logical technical alternative. The rationale of this method consists in the possibility of elevating the diaphragm to the desired level when it has been detached from the costodiaphragmatic recess, at the expense of a variable flattening of the dome. Fixing the diaphragm flap to the lowest healthy rib causes the immediate sealing of the pleura, eliminating most of the dynamic alterations of the wall secondary to the thoracectomy. Furthermore, the technique allows the diaphragm to be fixed on the patient’s intact ribs, avoiding both a diaphragm mesh and its direct anchoring onto the chest wall mesh. The diaphragm is not denervated by the phrenoplasty techniques since the fibers of the phrenic nerve branch outward from the center of the muscle, and at the same time, the vascular supply is not compromised. Thus, the fixed diaphragm retains muscle tone and some contractile functions, even though variable dome flattening and elevation can reduce excursion.

Tumors that require non-peripheral resections of the diaphragm close to the dome cannot be treated by phrenoplasty techniques since the condition for such procedures is that the muscle has kept the innervation and vascular supply intact.

This paper has not been designed as a case series analysis, and the small number of cases represents a limitation in terms of reproducibility and epidemiological impact. Indications for these procedures are very infrequent, and further experience from other high-volume centers is certainly needed.

## 5. Conclusions

The advantages of phrenoplasty are the following: (a) the thoracic or thoracoabdominal defect is changed into an abdominal one with reduced ventilatory impairment; (b) the diaphragm function is partially preserved because innervation and blood supply are spared, and the dome-like morphology resumes over time; (c) the paradoxical respiratory movements are completely avoided; (d) no further surgical trauma is added for reconstruction; (e) the procedure is quick and technically undemanding.

The disadvantages are related to the reduction in the pleural cavity volume, which varies according to the level of the rib to which the diaphragm is reattached.

## Data Availability

The data presented in this study are available on request from the corresponding author. The data are not publicly available due to patients’ privacy.
